# Editorial: Beneficial microbes for sustainable postharvest management of fresh produce

**DOI:** 10.3389/fmicb.2025.1639670

**Published:** 2025-06-20

**Authors:** Ajay Kumar, Manoj Kumar Solanki, Gustavo Santoyo

**Affiliations:** ^1^Amity Institute of Biotechnology, Amity University, Noida, Uttar Pradesh, India; ^2^Department of Life Sciences and Biological Sciences, IES University, Bhopal, Madhya Pradesh, India; ^3^Genomic Diversity Lab, Institute of Biological and Chemical Research, Universidad Michoacana de San Nicolás de Hidalgo, Morelia, Mexico

**Keywords:** postharvest management, microbial biocontrol in agriculture, fruit spoilage prevention, chemical-free preservation, postharvest innovations

Fresh produce, especially fruits and vegetables, is the richest source of nutrients, minerals, and vitamins, playing an essential role in ensuring food nutrition security worldwide. It is also one of the important commodities of global trade and economic development (Michel et al., 2024). According to estimates, the global annual farm gate value of fruits and vegetables is over USD 1 trillion, exceeding the total value of all agricultural food grains, which is only USD 837 billion. However, as a nutrient-rich source, the water and sugar content make fresh produce prone to pathogenic attack during growth and harvest or postharvest storage conditions. The Food and Agriculture Organization (FAO) estimated in 2024 that approximately 20%−40% of the fresh produce lost annually was due to phytopathogen attack. This loss raises concerns about ensuring food security for the rising global population (Schreinemachers et al., 2018).

To mitigate the challenges of phytopathogen attack, a large population relies on chemical pesticides; these work in a very short time, but their long-term use has shown detrimental effects on fruit quality, soil productivity, and environmental and human health. The last few years have shown an increase in consumer preference toward organic food or fresh produce grown with minimal use of chemical pesticides (Ambaye et al., 2024). In this context, the use of beneficial microorganisms that show antagonistic activity has been frequently employed as a microbial biocontrol agent to control phytopathogen growth or manage plant diseases during growth and harvest or under postharvest storage conditions. These microbial biocontrol agents are either used alone or in combination with other microbial strains of the same or different genera (syncoms) via different methods like seed treatment, plant or soil inoculants, foliar spray, or by dipping procedures (Yin et al., 2022).

The Research Topic “*Beneficial microbes for sustainable postharvest management of fresh produce*” has compiled six articles that cover various aspect of sustainable postharvest management, including screening of microbial biocontrol agents, evaluation of the biocontrol potential of metabolites, and how the microbial synthesized nanoparticles can take part in extending the shelf life of fresh produce under the postharvest storage conditions. For example, Li et al. isolated and characterized the bacterial strain *Bacillus megaterium*, which shows potential to degrade the aflatoxin B_1_ (AFB_1_) contamination from the Coix seed. The strains in the fermentation supernatant showed 97.45% degradation potential of AFB_1_ after 72 h at 57°C, with an initial pH of 8.0. These findings highlight the promising biocontrol potential of *B. megaterium* for mitigating AFB1 contamination in Coix seed.

Building upon the biocontrol role of *Bacillus* species, Du et al. reported the efficacy of *Bacillus tequilensis* against *Botrytis cinerea*, a gray mold pathogen responsible for significant postharvest losses in blueberries. The authors evaluated both the fermentation broth and the cell-free supernatant of *B. tequilensis* using biochemical and transcriptomic approaches, demonstrating notable antifungal activity.

In a related approach exploring natural compounds, Jiao et al. assessed the antifungal properties of paeonol, an active compound derived from traditional Chinese medicine, against *B. cinerea*. At a concentration of 250 mg/L, paeonol completely inhibited fungal growth. The study revealed its effectiveness in reducing gray mold and extending the shelf life and quality of cherry tomatoes, suggesting its potential as a natural antifungal alternative for postharvest storage.

Focusing on another major postharvest pathogen, Kuruppu et al. investigated the causal agents of black rot in pineapple across three different regions. *Thielaviopsis paradoxa* emerged as the primary pathogen, with a 45%−50% occurrence in two of the locations studied. The findings underscore the urgent need to develop effective control measures to protect pineapples from postharvest losses caused by *T. paradoxa*.

Nanotechnology-based strategies have also been explored for postharvest disease control. Kumawat et al. evaluated the antifungal potential of ZnO nanoparticles synthesized from *Serratia* sp. against *Aspergillus niger*, the causal agent of black mold in garlic. Treatment with 250 μg/mL ZnO-NPs suppressed mycelial growth by 90% and spore germination by 73%. Notably, a 500 ppm application in a pre-inoculation strategy resulted in 0% disease severity and significantly extended the postharvest shelf life of garlic.

Finally, in the context of microbial quality control in fruits, Tenea et al. explored bacterial postbiotics for preserving strawberries. The study employed postbiotic-based formulations (PBFs) that included antibacterial peptide-proteins from *Weissella cibaria* and exopolysaccharides from *W. confusa*. These formulations effectively reduced the viability of *Serratia liquefaciens*—a pathogen isolated from ready-to-eat strawberries—within 1 h, causing structural damage to bacterial cells and enhancing fruit shelf life and quality.

The articles compiled in this Research Topic help researchers in screening or formulating novel microbial biocontrol agents, nanoparticles, and metabolites, which can be used efficiently to reduce postharvest loss or enhance the shelf life of fresh produce in sustainable ways. We hope this Research Topic will provide a deep insight into postharvest management of fresh produce and related knowledge gaps.
